# Feeling abandoned but energized

**DOI:** 10.7554/eLife.106704

**Published:** 2025-03-25

**Authors:** Vaughn S Cooper

**Affiliations:** 1 Evolutionary microbiologist United States United States

**Keywords:** Science Under Threat in the United States, science, politics, science funding, careers in science, basic research, National Institutes of Health, None

## Abstract

Many individual researchers are frustrated by the response – or the lack of a response – from universities to a growing crisis.

The fact that this article contains a disclaimer – that I am writing in a personal capacity and not on behalf of my employer or institution – shows how academics have been left feeling handicapped in our efforts to oppose the Trump administration’s attacks on the National Institutes of Health (NIH) and science more broadly. Faced with existential threats to our careers and identities, we must defend alone or in Signal group chats, unlike the muscular, university-sanctioned protests of the past. It feels as if we are trying to fight a series of attacks on science with one arm tied behind our back and our lips half sewn shut.

We also feel alienated, abandoned, and underinformed because the general counsels of our universities have concluded, from their risk analyses, that hiding is better than fighting, even when they stare at the sword of Damocles. Instead, we tune into BlueSky, blogs or the mainstream media for updates. Will my grant be reviewed anytime this year? Will we accept graduate students? Will I have a job next semester? We are thankful for the heroes among us like Jeremy Berg who do the reporting and communicating that the institutional staff who are supposed to communicate with us are not doing.

Yet we are not beaten, and some of us are downright energized. All professional scientists are familiar with sacrifice and the need for abrupt course changes. It is true these are unprecedented attacks on American science that target its fundamental business model. We recognize that these attacks reflect a failure to fully communicate the benefits that federally and university co-funded research brings to the US in term of health, knowledge and global leadership. Every dollar invested returns two and a half to the economy, without counting priceless improvements in our daily lives. And every $1bn in cuts to NIH budget for extramural funding is likely to lead to the loss of at least 8000 jobs. We are deeply committed to protecting the nation’s critical biomedical infrastructure – its people – because their know-how is irreplaceable.

We know we must do better in justifying why we have chosen to become academic researchers and take below-market salaries to do so. In my own lab, we are eager to explain how our work – which has been stalled by the NIH freeze – could improve treatment of infections of catheterized patients or eliminate chronic wounds. As infectious disease researchers, we are accustomed to cycles of attacks and counterattacks, but these are usually attacks by pathogens that are evolving to evade immunity rather than attacks by politicians.

It is harder to handle the full-frontal assaults on the next generation of biomedical researchers. The freeze on graduate admissions at many institutions, combined with the elimination of scores of training programs that broaden access to careers in science, are wounding. Most of us are teachers and researchers, and they are taking our students away! This can lead us to lash out indiscriminately, even against our own communities, because the bleeding has not stopped. Add to this the scrubbing of DEI from the web pages of scientific societies and other ‘third place’ institutions – who are hiding from Project 2025 assaults on tax exemption or federal support – and it is easy to feel abandoned.

I find guidance in words from former NIH Director Elias Zerhouni: “Do not panic, and do not miss the rocks for the pebbles.” It is imperative that we identify the big issues and handle them in the right order. When we react, we must do so locally by engaging our representatives and having the people most affected alongside us. Many newly elected representatives do not yet have staffers with expertise in science or health, so we can help provide this guidance in meetings and correspondence as constituents. However, scientists must remember that we are less influential than the patients, farmers and students who our research aims to benefit. We should, therefore, build coalitions with other stakeholders to both lobby for science and rebuild public trust and respect for researchers. During this outreach, we must be generous and forgiving of anyone – peers, pundits, and policymakers alike – who may have hurt us out of misunderstanding or self-preservation. We must share the joy of discovery that motivated us to become and remain scientists as broadly and sincerely as we can.

Perhaps the most potent argument to build American research rather than cut it is the need to maintain our global leadership in science and technology. Government funding for research and development is just 0.7% of GDP, compared to 1.9% in 1964, and the US will soon fall behind China in total R&D. Americans from a range of professions believe the US has already ceded its global leadership in science, or will do so soon. They agree that the top obstacle to our future in science is the quality of our K–12 education in science, technology, engineering and mathematics. Given this need, what message is being sent to aspiring scientists by attacking the credibility and viability of the current and incoming cohort of researchers? That there is no future for them in science? We must avoid this tragedy at all costs. We scientists, colleges, organizations and societies owe it to our children that the US remains a sanctuary where one can dare to ask the biggest questions. Extinguishing this hope might be the most tragic loss of all.

